# Effect of Ripening Stage and Storage Temperature on the Survival and Internalization of *Salmonella* Typhimurium in ‘Maradol’ Papaya Fruit

**DOI:** 10.3390/foods15122170

**Published:** 2026-06-16

**Authors:** Mónica Cortés-Higareda, Rosa I. Ventura-Aguilar, Daniel Tapia-Maruri, Mónica Hernández-López, Patricia Landa-Salgado, Silvia Bautista-Baños

**Affiliations:** 1 Instituto Politécnico Nacional, Centro de Desarrollo de Productos Bióticos (CEPROBI), Carretera Yautepec-Jojutla, Km. 6, Calle CEPROBI 8, Colonia San Isidro, Yautepec C.P. 62739, Morelos, Mexico; mcortesh@yahoo.com (M.C.-H.); dmaruri@ipn.mx (D.T.-M.); mohernandezl@ipn.mx (M.H.-L.); 2 Departamento de Biotecnología, Universidad Autónoma Metropolitana-Iztapalapa, Av. Ferrocarril, San Rafael Atlixco 186, Colonia Leyes de Reforma, 1A Secc. Iztapalapa, México City C.P. 09310, Mexico; rventura@xanum.uam.mx; 3 Departamento de Ingeniería Agroindustrial, Universidad Autónoma Chapingo, Carretera Federal, Texcoco de Mora C.P. 56230, Estado de México, Mexico

**Keywords:** *Carica papaya* L., confocal laser scanning microscopy, environmental conditions, fruit quality, principal component analysis

## Abstract

Recent foodborne outbreaks by *Salmonella* Typhimurium associated with the consumption of ‘Maradol’ papaya have prompted an assessment of the pathogen’s behavior with regard to the fruit. The objective of this research was to assess the survival and possible internalization of *S*. Typhimurium in inoculated ‘Maradol’ papaya harvested at two maturity stages and stored for various days at 11 °C and 24 °C. The physicochemical analysis on fruit included firmness (N), total soluble solids (%), and pH. The location of *S.* Typhimurium in fruit exocarp and mesocarp tissues was observed using confocal laser scanning microscopy (CLSM). Results indicated that the high temperature (24 °C) was the most important factor for the survival of *S*. Typhimurium. At this same temperature, it was the variable firmness that was the most affected, regardless of storage days, whether inoculated or not. The principal component analysis (PCA) separated the data into two components that explained 57% of the variance. PC1 linked higher *S.* Typhimurium populations with declining firmness, TSS, and exocarp pH were found in fruit stored at 24 °C, whereas PC2 was associated with moderate physicochemical changes at 11 °C. The CLSM observations confirmed superficial colonization of the bacteria on papaya fruit rather than an internalization but only at 24 °C, regardless of ripening stage throughout the 7 days sampling period. Maintaining the cold chain is essential to mitigate the risk of salmonellosis in papaya.

## 1. Introduction

*Salmonella* spp. are Gram-negative rod-shape bacteria with remarkable genetic and phenotypic diversity and the ability to colonize a broad spectrum of hosts, including fresh horticultural commodities. They have been isolated from fruits and vegetables such as melon, cucumber, tomato, leafy greens, vegetable sprouts, strawberry, chili peppers, and papaya [1,2,3].

*Salmonella* infections generate substantial health problems and economic losses especially in those countries with limited health care systems. According to Kim et al. [4], direct medical costs per case range from approximately USD 546 to 22,000 for non-typhoidal *Salmonella* (NTS) and from USD 2000 to 33,000 for invasive NTS, varying by national income level. In Latin America, an estimated 77 million people fall ill annually from eating contaminated food with *Salmonella* being the cause, among other bacteria, of 95% of food-borne illnesses in this region [5].

Produce surveys report NTS prevalence levels ranging from 0.4% to 40% of the NTS, with serovars Typhimurium and Agona being the most frequently identified in fruits and vegetables in countries such as Mexico [5,6]. Berrocal et al. [7] identified four states with the highest incidence of *Salmonella* outbreaks linked to horticultural products such as papaya fruit (*Carica papaya*). Additional reports by the Food Drug Administration [8] and the Center for Disease Control and Prevention [9] have confirmed the presence of multiple *Salmonella* serotypes in ‘Maradol’ papayas imported to the United States. As reported by Whitney et al. [10] the contamination of whole fresh papayas has triggered several salmonellosis outbreaks in this country.

Papaya belongs to the family Caricaceae and is native to Mexico and Central America. Mexico is the world’s fourth largest exporter producing approximately 1.3 million tons in 2023 [11]. Throughout pre and postharvest handling, the fruit is exposed to numerous biotic (pathogenic microorganisms) and abiotic factors (including irrigation and wash water, mechanical damage, ripening, and storage temperature) as well as extensive human handling in order to prepare the fruit for the final market. These conditions can promote the survival, colonization, and internalization of *Salmonella* spp. [12,13].

He et al. [14] state that *Salmonella* employs several routes to internalize into host plants before and after harvest, entering through normal apertures such as the stomata and lenticels of plant organs such as roots, flowers, and fruit, and then moving passively within the vascular system via the transpiration stream. On this subject, Barak and Shroeder [15] demonstrated that contaminated irrigation water enabled *S. enterica* to colonize epidermal microcracks while engaging in the stomata of tomato fruit. In contrast, Kroupitski et al. [16] reported a chemotaxis-mediated internalization of *S. enterica* through stomata into the substomatal cavity of lettuce leaves. Burris et al. [17] highlighted the blossom-end internalization of cantaloupes by serovars Poona, Javiana, Newport, and Typhimurium via drip irrigation water. Environmental factors including rainfall, relative humidity (RH), pH, and storage temperature strongly modulate *Salmonella* survival and internalization [18].

As for papaya, Singh and Yemmireddy [12] inoculated diced fruit, with four serovar cocktails (Agona, Poona, St Paul, and Newport) observing that bacterial population increased with temperature, reaching their maximum levels at 21 °C. Furthermore, when the ripening stage was evaluated as a determinant of *Salmonella* survival, the greatest bacterial loads occurred on fully ripe fruit (100% yellow peel), under conditions of 21 °C and 90% RH [19].

Building on this background, the present study aimed to quantify the survival and observe internalization of *S*. Typhimurium in inoculated ‘Maradol’ papaya harvested at two ripening stages and stored for 7 days at either 11 °C or 24 °C. Relationships between bacterial dynamics and physiological changes in the fruit were explored through Principal Components Analysis (PCA). In addition, *S*. Typhymurium localization on papaya fruit was verified by the use of Confocal Scanning Microscopy (CSM).

## 2. Materials and Methods

### 2.1. Bacterial Strain

The strain of *Salmonella enterica* subesp. *enterica* serovar Typhimurium was obtained from the Postharvest Technology Laboratory of Agricultural Products (CEPROBI). Bacterial stocks were preserved at −80 °C in a trypticase soy broth (TSB, Bioxon, Mexico City, México) supplemented with sterile glycerol 1:1 (*v*/*v*).

A kanamycin-resistant strain was used throughout the study. For reactivation, frozen stocks were streaked onto Hektoen Enteric Agar (HEA; 76 g L^−1^, Bioxon, Mexico City, México), supplemented with kanamycin (50 µg mL^−1^), and incubated at 37 °C for 24 h. A single colony was transferred to 10 mL of TSB supplemented with kanamycin (50 µg mL^−1^) and incubated at 37 °C for 24 h with orbital shaking at 50 rpm (Innova 40, Edison NJ, USA) [20].

### 2.2. Preparation of S. Typhimurium

The kanamycin-resistant *S.* Typhimurium strain was streaked on HEA-Km^50^ agar (HEA supplemented with kanamycin, 50 µg mL^−1^) and incubated at 37 °C for 48 h. A single colony was inoculated into 50 mL of TSB-Km^50^ (TSB supplemented with kanamycin, 50 µg mL^−1^) in a 100 mL Erlenmeyer flask and incubated for 24 h at 37 °C with orbital shaking at 50 rpm. Cells were harvested by centrifugation (Prism Microcentrifuge, Labnet, Edison NJ, USA) at 8000× *g* for 10 min, at 24 °C, washed twice with sterile distilled water, and resuspended in 1 mL of sterile distilled water. The bacterial suspension was maintained at 11 °C until further use. A working inoculum of 8 Log_10_ CFU mL^−1^ was prepared by serial ten-fold dilutions (1:9) in sterile peptonized water (1.0% *w*/*v*), and cell concentration was verified spectrophotometrically at 546 nm (OD_546_ = 0.75).

### 2.3. Fruit Material

‘Maradol’ papayas were harvested in Yautepec Morelos, Mexico at two ripening stages: 50% and 75% of yellow peel coloration. Fruit were transported in plastic containers to the Postharvest Technology Laboratory of Agricultural Products in CEPROBI.

### 2.4. Experimental Conditions and Treatments

Fruit were quickly washed with neutral soap, rinsed with sterile distilled water, immersed for 1 min in 1% (*v*/*v*) sodium hypochlorite solution, rinsed again, dipped in 70% ethanol, rinsed with distilled water, and air-dried.

For the inoculated treatments, 100 µL of the *S.* Typhimurium suspension previously prepared (8 Log_10_ CFU mL^−1^) was applied in five drops (≈2 cm^2^ total area) onto a previously marked place area of the exocarp. The control groups received 100 µL of sterile distilled water on the marked areas of the fruit. After air-drying papayas at ambient temperature, they were stored either in cold conditions (11 °C ± 2, 90% RH) or at ambient temperature (24 °C ± 2, 46% RH). For evaluation, exocarp (peel) and mesocarp (pulp) tissues were sampled on days 3, 5, and 7. For maintaining the RH in the cool room, two 10 L plastic containers fill with water were placed inside, whereas at room temperature it was not controlled. The RH was constantly measured using a digital hygro-thermometer clock (EXTECH Instruments, Hudson, NH, USA).

Eight treatments (each in triplicate) were established: T1 = inoculated papaya at 50% ripening stage and stored at 11 °C, T2 = non-inoculated papaya at 50% ripening stage and stored at 11 °C, T3 = inoculated papaya at 50% ripening stage and stored at 24 °C, T4 = non-inoculated papaya at 50% ripening stage and stored at 24 °C, T5 = inoculated papaya at 75% ripening stage and stored at 11 °C, T6 = non-inoculated papaya at 75% ripening stage and stored at 11 °C, T7 = inoculated papaya at 75% ripening stage and stored at 24 °C, T8 = non-inoculated papaya at 75% ripening stage and stored at 24 °C. Five fruit were used per replicate.

### 2.5. Isolation of S. Typhimurium from Inoculated Papaya Fruit

One g of exocarp or mesocarp tissue was homogenized in 9 mL of 1% peptonized water. Decimal dilutions (10^−1^ to 10^−6^) were spread (100 µL) onto HEA-Km^50^ agar and incubated at 37 °C for 24 ± 2 h. Counts were expressed as log_10_ CFU g^−1^ [21].

### 2.6. Physical and Chemical Characteristics of Papaya Fruit

The firmness on the equatorial region of each fruit was measured twice with a penetrometer (Lutron FR-5120, Coopersburg, PA, USA). The two readings were averaged and expressed in Newtons (N). For pH determination, 10 g samples of exocarp and mesocarp tissue were measured using a benchtop pH meter (Orion Versa Star Pro. Thermo Scientific, Nashua, NY, USA). The total soluble solids content (TSS) was measured in the mesocarp juice with a handheld refractometer (N-1E, Atago, 0–32% °Brix scale, Minato-ku Osaka, Japan). The results were reported as percentage of total soluble solids (TSSs).

### 2.7. Observations of S. Typhimurium by Confocal Laser-Scanning Microscopy (CLSM)

Fresh, hand-cut cross-sections of the papaya exocarp and mesocarp (approximately ≈12 mm × 10 mm × 4 mm) were obtained using a sterile sharp razor blade. The tissue samples were placed on microscope glass slides and immediately stained with the respective fluorophores as follows:

1º.—acridine orange (a specific fluorochrome to detect *S*. Typhimurium viable cells), prepared at 1 mg 50 mL^−1^ in phosphate-buffered saline (PBS), incubated for 25 min at dark, and rinsed with PBS. 2º.—in propidium iodide, (fluorochrome to highlight the plant cell architecture), prepared at 1 mg 50 mL^−1^ in PBS, incubated under the same conditions, and rinsed again with PBS.

After 1 h, the stained samples were coverslipped and examined using a confocal laser scanning microscope (CLSM) (LSM 800, Carl Zeiss, Oberkochen, Germany) at 400× magnification. The fluorescence intensity was quantified with the use of ZEN 2.3 Blue software [22].

### 2.8. Statistical Analysis

The physicochemical assays were analyzed using a two-way ANOVA experiment with factors, including types of tissue and inoculation, storage temperature, and storage days, in a completely randomized design with five replications per treatment combination. The Fisher’s Least Significant Difference (LSD) test was used to compare means (*p* ˂ 0.05). In addition, to explore correlations among ripening stages, storage temperature, physicochemical attributes, and *S.* Typhimurium CFU, in the exocarp and mesocarp tissue of papayas, data were analyzed using a Principal Component Analysis (PCA). The INFOSTAT^®^ 2020 statistical software was employed for both tests. The PCA obtains information from the statistical data by representing it as a set of new orthogonal variables called principal components that are composites or blends of the original variables and displays the patterns of similarity between the observations and the variables as points on spot maps and in a correlation matrix [23].

## 3. Results

### 3.1. Survival of S. Typhimurium in Papaya Fruit

In this study, overall, *S*. Typhimurium survival on inoculated papaya fruit depended mainly on storage temperature and ripening stage (Table 1). For instance, in papaya fruit stored at 11 °C, regardless of papaya tissue, ripening stage, and storage days, no bacterial growth was observed. On the contrary, at 24 °C, only on the fruit exocarp at ripening of 75% and in the storage days, the CFU was from 2.2 to 2.5 log10 CFU g-1 (*p* < 0.05).

Coinciding with these results, previous studies have concluded that the temperature is one the main factors influencing *Salmonella* growth on various horticultural products. For example, on tomatoes, the *Salmonella* population increased more rapidly at 21 °C than at 12 °C, regardless of the variety and ripening stage [24]. Similarly, storage temperature outweighed the RH and nutrient effect [25]. For six leafy greens, such as iceberg lettuce, romaine lettuce, red lettuce, green onion, spinach, and kale, storage at 25 °C produced the highest cell count of Salmonella [14], while Saha et al. [26] observed an analogous pattern in the case of Kate mango, with maximal Salmonella growth at 30 °C relative to 12 °C and 20 °C.

In this research, *S*. Typhimurium inoculum did not accelerate the overall ripening of the papaya fruit. The storage behavior of the inoculated ones was similar to those non-inoculated. Papaya fruit being a climacteric fruit faces a very short storage life. Therefore, the observed physicochemical changes except for the storage temperature could be more due to their climacteric behavior and the intrinsic particular metabolism of papayas than to the other tested factors [27].

### 3.2. Physicochemical Properties of the Inoculated ‘Maradol’ Papaya

With respect to physicochemical variables, there were significant differences (*p* < 0.05) among the evaluated factors (Table 2). However, the storage temperature and ripening stage were the main important factors influencing fruit firmness, regardless of the tissue type, storage duration, and inoculated condition. In general, fruit firmness decreased with storage time. At the end of the seven days storage period at 11 °C, the final value was close to 30 N in fruit harvested at 50% ripening stage, at both temperatures regardless of inoculation treatment. In comparison, at 24 °C, the average fruit firmness values ranged from 12 N to 18 N. The fruit TSS did not follow any consistent pattern with respect to the remaining tested factors, showing values from 8.3% to 13% at the end of storage. Likewise, exocarp and mesocarp pH remained relatively stable with respect to the harvest value (6.2), regardless of storage temperature and storage period, ripening stage, and whether inoculated or not.

### 3.3. PCA

According to Terrádez [28] there is no definite rule regarding the number of principal components (PCs) to be used; however, one of the objectives of the PCA is dimensionality reduction. Therefore, practical applications often aim for at least 50–70% of total variance to be explained by the first few PCs.

In this study, specifically, PC1 represented the physicochemical ripening gradient of papaya associated with changes in firmness, TSS, and pH, whereas PC2 reflected variability related to *S*. Typhimurium survival patterns and their relationship with the physicochemical characteristics of fruit under different storage temperatures.

**Figure 1:**
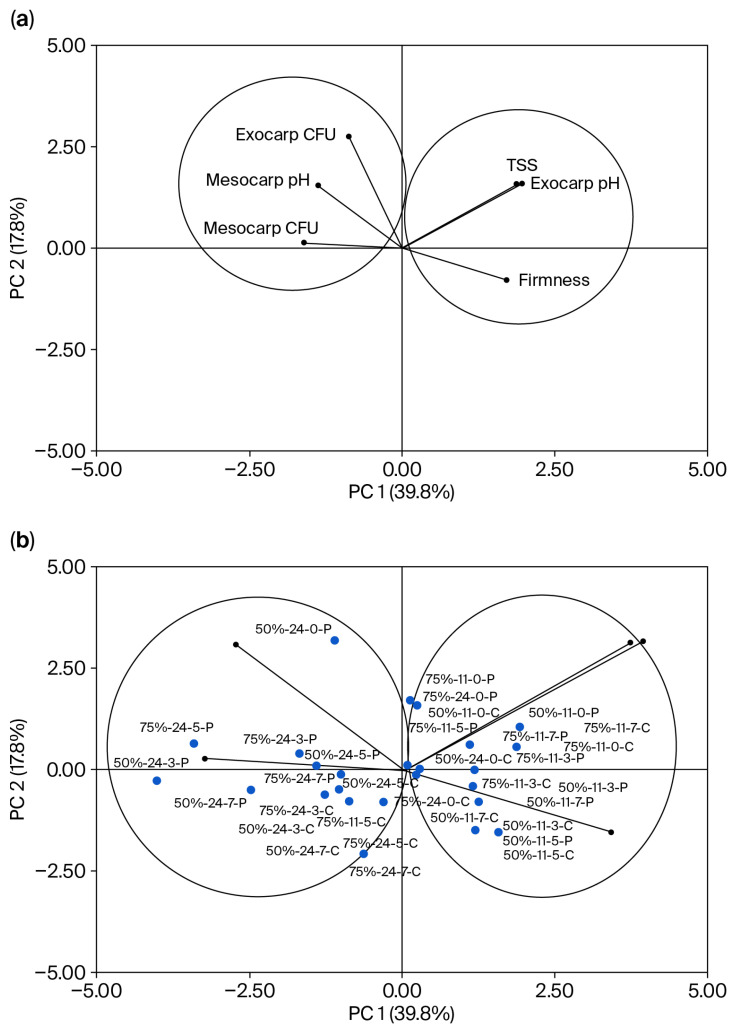
Principal component analysis (PCA) summarizing physicochemical attributes of *Salmonella* Typhimurium populations in ‘Maradol’ papaya. Fruit were harvested at two ripening stages (50% and 75% yellow peel) and stored for seven days at either 11 °C or 24 °C. (**a**) score plot of PC1 (39.8% variance) versus PC2 (17.8% variance) showing treatment groupings. (**b**) loading plot indicating the contributions of firmness, TSS, exocarp pH, mesocarp pH, and bacterial counts (log_10_ CFU g^−1^) in exocarp and mesocarp to each principal component. Data in (**b**) were labeled according to ripening stage (50% and 75%), storage temperature (11 °C and 24 °C), storage days (3, 5, and 7), and the type of tissue analyzed (P = mesocarp and C = exocarp).

The PCA reduced the nine monitored variables into two PCs explaining 57.6% of the total variance (PC1 = 39.8%, PC2 = 17.8%). Regardless of ripening stage, papayas stored at 11 °C clustered along PC2, where *S.* Typhimurium populations were positively associated with TSS and with pH of exocarp and mesocarp but negatively associated with firmness (Figure 1a). In contrast, fruit stored at 24 °C clustered along PC1 where bacterial populations had negative correlations with exocarp pH, TSS, and firmness (Figure 1b). The relatively weak correlations observed between microbiological variables in exocarp and mesocarp and the physicochemical characteristics, compared with the stronger associations among the physicochemical variables, suggest that additional factors may influence *S*. Typhimurium behavior on papaya. Although this bacterium possesses acid tolerance mechanisms allowing survival under acidic conditions, its optimal growth generally occurs under near-neutral pH values (approximately pH 6.5–7.5), consistent with neutrophilic growth behavior [29]. Large diameter porins outer membrane facilitate protons diffusion and could alter homeostasis [30]. Conversely, a negative relationship between pH and *Salmonella* populations has been documented for strawberries, blueberries, and tomatoes but not in papaya [31]. Nevertheless, Beuchat and Mann [24] reported that several serovars (Agona, Baildon, Gaminara, Michigan, and Montevideo) grew on tomatoes at both 1 °C and 21 °C despite low pH and high °Brix values, suggesting commodity-specific responses.

In this study, the inverse association between TSS and bacterial populations could be related to an increase in osmotic pressure, usually caused by TSS, which in turn could have reduced water activity and consequently, bacterial fitness [32]. Likewise, firmer fruit likely provide fewer microenvironments and damaged tissue available for bacterial attachment, explaining the negative correlation between firmness and bacterial populations [33]. The temperature remained the predominant factor driving bacterial proliferation, with higher storage temperatures consistently associated to greater pathogen loads, in line with reports for lettuce, mango, and other produces [3,25]. To the best of our knowledge, this is the first study to integrate fruit quality parameters with *S.* Typhimurium behavior in ‘Maradol’ papaya through PCA, providing a multivariate perspective on the factors influencing the bacterium survival and probable internalization.

### 3.4. Confocal Laser-Scanning Microscopy (CLSM) of Exocarp and Mesocarp Tissues of ‘Maradol’ Papaya

**Figure 2:**
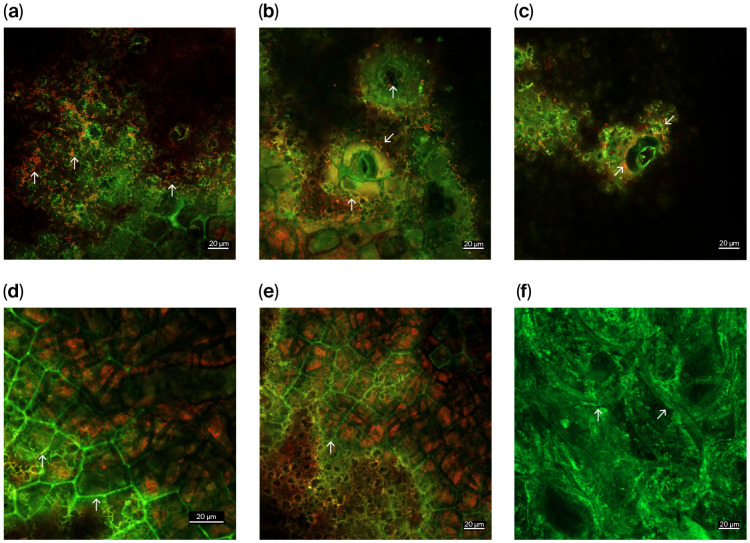
Confocal laser scanning microscopy (CLSM) micrographs of ‘Maradol’ papaya inoculated with *Salmonella* Typhimurium at different ripening stages, storage days, and conditions. General description: Scale bars represent 50 µm. Arrows indicate the presence of red-orange fluorescent rods characteristic of *S.* Typhimurium and stomata and parenchyma cells of papaya fruit (green fluorescent). (**a**) Exocarp (50% ripe). Fruit stored for 3 days at 24 °C. Bacterial cells are observed on the fruit surface. (**b**) Exocarp (75% ripe). Fruit stored for 3 days at 24 °C. Bacterial density is also observed surrounding and attached to the stomata. (**c**) Exocarp (75% ripe). Fruit stored for 7 days at 24 °C. Abundant bacterial cells persist associated with the stomatal area and on fruit surface. (**d**) Exocarp (50% ripe). Fruit stored for 7 days at 11 °C; No presence of *S*. Typhimurium, only parenchyma cells are observed (green fluorescent). (**e**) Exocarp (75% ripe). Fruit stored for 7 days at 11 °C. No presence of *S*. Typhimurium. Only parenchyma cells are observed (green fluorescent). (**f**) Mesocarp (pulp). Overall representative image of parenchyma tissue (green fluorescent) at all storage conditions, confirming the absence of *S*. Typhimurium in this tissue.

The CLSM observations provided valuable visual evidence through the clear description of bacterial localization shown in Figure 2a–c. Under CLSM, *S*. Typhimurium cells appeared as elongated red-orange fluorescing rods (<100 µm). On the exocarp of fruit harvested at 50% yellow peel stage and at 24 °C storage temperature, bacterial cells were already visible by day three (Figure 2a). In 75% yellow harvested fruit, stored at the same temperature, bacterial density was high on both sampling days (three and seven). Overall, the bacterial cells were concentrated and associated with stomata, which fluoresced bright green in color (Figure 2b,c). The stomata cells (two guard cells) were observed spreading out on the fruit epidermis. No bacterial cells were detected on papayas stored at 11 °C at either ripening stage (Figure 2d), nor were they observed in either the mesocarp of any treatment, inoculated, and control fruit (Figure 2e,f).

In this study, under the experimental condition given, the bacterial behavior suggested colonization rather than internalization. On this subject, Pérez-Lavalle et al. [34,35] likewise reported surface colonization of strawberries by *S*. Thompson after storage at 20 °C for 3 days and refrigerated at 4 °C to 7 °C for 10 days, describing comparable shaped fluorescent cells; however, these authors observed biofilm aggregates on refrigerated fruit, whereas in our papayas, there was only an increase in cell numbers with no visible aggregation. Our results could be attributed to the natural antibacterial properties of papaya fruit.

These findings may be partially associated with the antimicrobial properties of papaya fruit. Although there are no available reports specifically addressing *S.* Typhimurium interactions with papaya, it has been documented the inhibitory activity of papaya latex and solvent extracts against diverse bacteria including *Escherichia coli*, *Staphylococcus aureus*, and *Pseudomonas aeruginosa* [36,37].

Furthermore, natural defenses mechanisms, together with structural barriers including the cuticle and wax layer components, could limit pathogen survival or tissue penetration on intact fruit surfaces [38]. For example, Islam et al. [39] demonstrated that papaya latex inhibited bacterial growth almost as effectively as the reference antibiotic ciprofloxacin (50 µg ciprofloxacin) with *E. coli* being particularly sensitive. Similarly, Egbuonu et al. [36] reported antimicrobial activity of ethanolic extracts of papaya peel (10 mg mL^−1^) which produced inhibition zones of 15 mm, 17.3 mm, and 14.3 mm against *Staphylococcus aureus*, *E. coli*, and *Pseudomonas aeruginosa*, respectively, whereas no inhibition was observed in the control.

These findings suggest that *Salmonella* outbreaks associated with ‘Maradol’ papaya are more likely due to cross-contamination during postharvest handling than to in-field proliferation. As Collignon and Korsten [40] emphasize, foodborne pathogens must first reach the fruit surface, attach, and proliferate to levels exceeding the infectious threshold at which illness can occur.

## 4. Conclusions

The persistence of *S*. Typhimurium on ‘Maradol’ papaya was primarily influenced by storage temperatures. Except for fruit firmness in papayas stored at 24 °C, the ripening- related characteristics such as TSS content and pH were not substantially affected by the evaluated factors. PCA reduced the studied variables into two components that together explained 57.6% of the total variance (PC1 = 39.8%, PC2 = 17.8%). At 11 °C, no bacterial populations were recorded in either exocarp or mesocarp tissues during the 7 days trial. In contrast, the storage at 24 °C favored its survival throughout the sampling period. The CLSM observations provided valuable visual evidence of bacterial localization during this period and an effective comparison with previous studies. Although at 24 °C, these bacterial cells were observed largely confined to surface structures and stomatal regions, its internalization was not demonstrated towards the internal mesocarp tissue of fruit during the 7-day storage period. The limitations of this study, including the use of artificial inoculation and the relatively short storage duration, should be considered for future studies addressing *S*. Typhimurium internalization. Nevertheless, the storage temperature and ripening stage emerge as key control points influencing its behavior. Consequently, strict cold-chain management during handling and storage would be essential to prevent *S*. Typhimurium and associated outbreaks.

## Figures and Tables

**Table 1:** *Salmonella* Typhimurium survival in exocarp and mesocarp tissue on inoculated and non-inoculated ‘Maradol’ papaya, harvested at two ripening stages, and stored at different temperatures and days.

Papaya Tissue	Initial Presence of *S*. Typhimurium	Ripening Stage at Harvest	Storage Temperature	Storage Days	*Salmonella* Typhimurium (Log_10_ CFU g^−1^)
Exocarp	Inoculated and non-inoculated	50%	11 °C	3, 5, 7	0
Mesocarp	Inoculated and non-inoculated	50%		3, 5, 7	0
Exocarp	Inoculated and non-inoculated	75%	11 °C	3, 5, 7	0
Mesocarp	Inoculated and non-inoculated	75%		3, 5, 7	0
Exocarp	Inoculated and non-inoculated	50%	24 °C	3, 5, 7	0
Mesocarp	Inoculated and non-inoculated	50%		3, 5, 7	0
Exocarp	Inoculated	75%	24 °C	3	2.2 ± 0.3 ^a^
	Inoculated			5	2.5 ± 0.4 ^a^
	Inoculated			7	2.4 ± 0.2 ^a^
Exocarp	Non-inoculated	75%	24 °C	3, 5, 7	0
	Inoculated and non-inoculated			3, 5, 7	0

Initial *S.* Typhimurium concentration = 8 log_10_ CFU g^−1^. Means with common letters are not significantly different (LSD test; *p* < 0.05).

**Table 2:** Changes in firmness, TSS, and pH of inoculated or not ‘Maradol’ papaya with *Salmonella* Typhimurium, harvested at two ripening stages, and stored under different temperatures during seven days.

Storage Temperature and Inoculum	Storage Days	Ripening Stage		Ripening Stage		Ripening Stage		Ripening Stage	
		50%	75%	50%	75%	50%	75%	50%	75%
		Firmness (N)		TSS (%)		pH (Fruit Exocarp)		pH (Fruit Mesocarp)	
11 °C Inoculated	3	29.8 ± 3.2 aA *	21.8 ± 3.1 bB	10.3 ± 1.1 bA	10.5 ± 2.7 aA	5.5 ± 0.01 aA	5.7 ± 0.1 aA	5.8 ± 0.1 aA	5.9 ± 0.1 aA
	5	29.6 ± 1.5 aA	18.6 ± 0.9 aB	8.7 ± 1.5 bA	9.7 ± 0.5 bA	5.5 ± 0.01 aA	5.4 ± 0.1 bA	5.7 ± 0.2 aA	5.9 ± 0.1 aA
	7	29.9 ± 2.4 aA	18.9 ± 1.6 aA	10 ± 1.3 bA	10.2 ± 0.5 aB	5.5 ± 0.1 aA	5.6 ± 0.1 aB	5.7 ± 0.1 aA	6 ± 0.1 aA
11 °C Non-inoculated	3	31.5 ± 3.6 aA	28.4 ± 3.6 aA	12.7 ± 1.5 abA	11 ± 0.1 aA	5.6 ± 0.1 aA	5.7 ± 0.1 aA	5.6 ± 0.02 aA	6.1 ± 0.1 aB
	5	32.6 ± 4.8 aA	18.6 ± 4.6 aB	8.7 ± 0.5 bA	10.5 ± 0.4 aB	5.4 ± 0.1 aA	5.5 ± 0.2 bA	5.8 ± 0.08 aA	6 ± 0.1 aB
	7	29.9 ± 4.8 aA	17.9 ± 1.3 aB	11.2 ± 0.7 aA	12 ± 1 cA	5.5 ± 0.1 aA	5.6 ± 0.07 aA	5.8 ± 0.2 aA	6.1 ± 0.2 aA
24 °C Inoculated	3	24 ± 0.2 abA	14.8 ± 1.6 bB	9.6 ± 2 bA	8.3 ± 0.2 bA	5 ± 0.2 bA	5.4 ± 0.08 abA	6.3 ± 0.1 bA	6.1 ± 0.1 aA
	5	16.8 ± 3.2 cA	21.6 ± 3.6 bA	10.1 ± 1.3 bA	8.7 ± 1.1 bA	5.3 ± 0.08 cA	5.4 ± 0.1 bA	5.9 ± 0.1 aA	6 ± 0.1 aA
	7	12.3 ± 3.5 cA	25.4 ± 3.2 aB	9.8 ± 0.2 bA	7.7 ± 1.5 bA	5.1 ± 0.09 bA	5.0 ± 0.1 cA	5.9 ± 0.2 aA	5.9 ± 0.2 aA
24 °C Non-inoculated	3	12.6 ± 2.3 cA	15.4 ± 1.9 bA	7.2 ± 0.7 cA	8.7 ± 0.5 bA	5.1 ± 0.2 bA	5.3 ± 0.2 bA	6.2 ± 0.1 bA	6.2 ± 0.1 aA
	5	15.4 ± 4.5 cA	16.9 ± 2.6 bA	8.9 ± 1.1 bA	8.2 ± 0.7 bA	5.2 ± 0.4 cA	5.1 ± 0.1 cA	6.6 ± 0.8 cA	6.1 ± 0.1 aA
	7	18.5 ± 4.9 cAA	18.9 ± 2.8 bA	8.9 ± 0.9 bA	9.7 ± 2 bA	4.8 ± 0.3 bA	5.1 ± 0.3 cA	6.6 ± 0.3 b	5.9 ± 0.4 a

Initial firmness = 20–28.6 N, TSS = 9.6–11.3%, exocarp pH = 5.4–5.7, mesocarp pH = 6.0–6.5. * Lowercase and capital letters indicate significant differences by LSD test (*p* ˂ 0.05) among rows (temperature and inoculation) and columns (ripening stages), respectively.

## Data Availability

The original contributions presented in this study are included in the article. Further inquiries can be directed to the corresponding authors.
